# The Yeast ATF1 Acetyltransferase Efficiently Acetylates Insect Pheromone Alcohols: Implications for the Biological Production of Moth Pheromones

**DOI:** 10.1007/s11745-016-4122-4

**Published:** 2016-01-22

**Authors:** Bao-Jian Ding, Ida Lager, Sunil Bansal, Timothy P. Durrett, Sten Stymne, Christer Löfstedt

**Affiliations:** Department of Biology, Lund University, Lund, Sweden; Department of Plant Breeding, Swedish University of Agricultural Sciences, Alnarp, Sweden; Department of Biochemistry and Molecular Biophysics, Kansas State University, Manhattan, USA

**Keywords:** Acetyltransferase, Fatty alcohol, Acetates, Moth pheromone, Yeast expression

## Abstract

Many moth pheromones are composed of mixtures of acetates of long-chain (≥10 carbon) fatty alcohols. Moth pheromone precursors such as fatty acids and fatty alcohols can be produced in yeast by the heterologous expression of genes involved in insect pheromone production. Acetyltransferases that subsequently catalyze the formation of acetates by transfer of the acetate unit from acetyl-CoA to a fatty alcohol have been postulated in pheromone biosynthesis. However, so far no fatty alcohol acetyltransferases responsible for the production of straight chain alkyl acetate pheromone components in insects have been identified. In search for a non-insect acetyltransferase alternative, we expressed a plant-derived diacylglycerol acetyltransferase (EaDAcT) (EC 2.3.1.20) cloned from the seed of the burning bush (*Euonymus alatus*) in a yeast system. EaDAcT transformed various fatty alcohol insect pheromone precursors into acetates but we also found high background acetylation activities. Only one enzyme in yeast was shown to be responsible for the majority of that background activity, the acetyltransferase ATF1 (EC 2.3.1.84). We further investigated the usefulness of *ATF1* for the conversion of moth pheromone alcohols into acetates in comparison with *EaDAcT*. Overexpression of *ATF1* revealed that it was capable of acetylating these fatty alcohols with chain lengths from 10 to 18 carbons with up to 27- and 10-fold higher *in vivo* and *in vitro* efficiency, respectively, compared to *EaDAcT*. The ATF1 enzyme thus has the potential to serve as the missing enzyme in the reconstruction of the biosynthetic pathway of insect acetate pheromones from precursor fatty acids in yeast.

## Introduction

Moth species rely heavily on volatile sex pheromones for mate finding. More than 600 moth pheromones have been identified and a substantial portion of these contain acetates of fatty alcohols [1]. During the last three decades, pheromone biosynthesis pathways have been studied intensively in many moth species and great progress has been achieved in understanding the biosynthesis of these volatile compounds. The genes coding for the fatty acyl desaturases (FAD) that introduce double bonds into the acyl chain and the fatty acyl reductases (FAR) that convert the fatty acids into fatty alcohols have been characterized [2–9]. The acetylation of fatty alcohols is the last step in the biosynthesis of many moth pheromones. While *in vivo* labeling experiments have been performed and the substrate specificity studied *in vivo* [10, 11], no enzyme catalyzing this reaction has been identified and cloned from any insect species. The acetyltransferases involved in moth pheromone production probably belong to a large family of acyl CoA-utilizing enzymes which produce a variety of products, including neurotransmitters, volatile esters, constitutive defense compounds, waxes, phytoalexins, lignin, phenolics, alkaloids, and anthocyanins [12–18], making it very difficult to make functional predictions from primary sequences alone. Indeed, attempts to identify the acetyltransferase from insects have only resulted in enzymes that acetylate other compounds [19].

Moth pheromones can be produced in biological systems for use in pest control. We have recently explored the possibility of producing pheromones in plants [20] as well as in microbial cell factories [21]. The lack of a fatty alcohol acetyltransferase represents a bottleneck in our attempts to produce moth pheromone compounds biologically. Previously, to produce moth pheromone acetates in *Nicotiana benthamiana* by transient expression, we used the acetyltransferase *EaDAcT* cloned from *Euonymusalatus* (burning bush), but the efficiency was low [20]. In the present study, we investigate the substrate specificity of the plant-derived acetyltransferase EaDAcT when expressed in *Saccharomyces cerevisiae* for the acetylation of moth pheromone alcohols. In doing so, we uncovered the ability of the yeast acetyltransferase ATF1 to also acetylate these alcohols. ATF1 is one of three known *S. cerevisiae* alcohol acetyltransferases responsible for the synthesis of volatile esters that contribute to the fruity aroma of fermented alcoholic beverages [22]. Deletion or overexpression of these genes confirmed their roles in the formation of a broad range of short-chain acetates from propyl acetate up to octyl acetate [22]. It has recently been shown that ATF1 can also acetylate saturated fatty alcohols with a chain length of 14C and 16C when expressed in *Escherichia coli* [23, 24]. Here, we use both *in vivo* and *in vitro* assays to compare the efficiency of the acetyltransferases for acetylation of various fatty alcohols.

## Materials and Methods

### Yeast *In Vivo* Assay

*EaDAcT* and *ATF1* genes were cloned into the galactose-inducible pYES-DEST52 yeast expression vector by the Gateway cloning system (Invitrogen). After confirmation of the constructs by sequencing, they were transformed into wild-type or *atf1∆* yeast strains. For selection of uracil prototrophs, the transformed yeast was allowed to grow on SC-U selective plates containing 0.7 % YNB (without amino acids, with ammonium sulfate), amino acid drop-out lacking uracil (Formedium™ Ltd, Norwich, England), and 2 % glucose. After 2 days incubation at 30 °C, individual colonies were picked to inoculate 2 mL selective medium at 30 °C and grown at 300 rpm for 48 h. Yeast cultures were diluted to an OD600 of 0.4 in 2 mL fresh selective medium (1 % Tergitol, type Nonidet NP-40, Sigma-Aldrich) containing 2 % galactose, supplemented with the fatty alcohol to be tested at 0.5 mM final concentration, and incubated at 300 rpm for 48 h at 30 °C (three replicate cultures). Cells and medium were separated by centrifugation at 4000 rpm for 2 min. Acetates in the media were extracted with 800 µL of hexane [with internal standard (*Z*)-8-tridecenyl acetate (Z8-13:OAc), 0.25 ng/µL], the extract was washed with water and subsequently analyzed by GC–MS. Cells were extracted with 800 µL of hexane (with internal standard Z8-13:OAc, 0.25 ng/µL) plus sonication. After brief centrifugation, the supernatant was transferred to a new tube and then analyzed by GC–MS.

GC–MS analyses were performed on a Hewlett Packard 6890 GC coupled to an HP 5973 mass-selective detector. The GC was equipped with an INNOWax column (30 m × 0.25 mm × 0.25 µm), and helium was used as carrier gas (average velocity 33 cm/s). The MS was operated in electron impact mode (70 eV), scanning between *m/z* 30 and 400, and the injector was configured in splitless mode at 220 °C. The oven temperature was set to 80 °C for 1 min, then increased at a rate of 10 °C/min to 210 °C, followed by a hold at 210 °C for 15 min, and then increased at a rate of 10 °C/min to 230 °C followed by a hold at 230 °C for 20 min. Compounds were identified by comparison of retention times and mass spectra with those of reference compounds available in our laboratory collection. Compounds were quantified by the total ion current recorded.

### Yeast Microsomal Activity Assay

Yeast strains harboring the pYES-*EaDAcT* and pYES-*ATF1*, respectively, were grown in 250 mL SC-U medium in 1 L flasks at 30 °C with shaking 200 rpm for 48 h. Yeast cells were pelleted in 50 mL tubes by spinning at 2000*g* for 5 min at 4 °C and washed with 20 mL ice cold 20 mM Tris–HCl (pH 7.9), and suspended in 1 mL Tris–HCl buffer. The cells were transferred to 2 mL screw-capped tubes, pelleted down, and the supernatant removed. Silica/zirconium beads (0.5 mm diameter; 1 mL) were added to the tubes and the remaining space filled with disruption buffer [20 mM Tris–HCl, pH 7.9, 10 mM MgCl_2_, 1 mM EDTA, 5 % (v/v) glycerol, 1 mM DTT, 0.3 M ammonium sulfate]. The cells were disrupted using a Beadbeater (MP FastPrep-24) for eight periods of 30 s, with 30 s rests in between periods. The broken cells were transferred to a 50 mL tube and the volume was adjusted to 20 mL with disruption buffer and centrifuged at 2000*g* to remove the beads. The supernatant was transferred to a new tube and centrifuged at 10,000*g* for 10 min at 4 °C to remove the cell debris and large organelles. The supernatant containing the membrane and associated proteins were centrifuged at 100,000*g* at 4 °C for 2 h. The obtained microsomes were dissolved in 1 mL resuspension buffer [50 mM Tris–HCL pH 7.9, 20 % (v/v) glycerol, 1 mM DTT]. The microsomal membranes of the yeast expressing the *EaDAcT* gene and *ATF1* gene were prewashed with acetone to remove the diacylglycerols that would otherwise compete with fatty alcohols. To remove the DAG associated with the membrane, 1 mL of microsomal membrane was added to 50 mL of cold (−80 °C) acetone under gentle magnetic stirring in the cold room (4 °C) for 10 min [25]. The clean membranes were resuspended in water and stored in aliquots at −80 °C before being used for enzyme assays. Protein concentration was quantified using the BCA method (Thermo Scientific).

About 150 µg of microsomal protein was used for the acetyltransferase activity assay in a total volume of 100 µL containing 15 µL of a fatty alcohol (120 nmol in DMSO), 5 µL (50 nmol) of [^14^C]acetyl-CoA (3000 dpm/nmol) and 60 µL of reaction buffer [50 mM HEPES pH 7.4, 10 % (v/v) glycerol, 5 mM MgCl_2_, 1 mM DTT] (two replicate assays). After incubation at 30 °C for 40 min, the reaction was stopped by adding 500 µL MeOH/CHCl_3_ (1:1, by vol) and 100 µL 0.15 M HAc with vortexing. The chloroform phase was separated by a low speed centrifugation and divided into two parts: 1/5 was subjected to scintillation counting whereas 4/5 was loaded on a silicagel 60 TLC plate (Merck) and developed in a 70:30:1 (v/v/v) heptane/diethylether/HAc solvent system. The percentage of radioactivity in each band was determined with electronic autoradiography using an Instant Imager (Canberra Packard) electronic autoradiograph and the amount of product formed was calculated using the scintillation count. In case of duplicate samples, the variation between samples is not depicted in the graphs but values never deviated more than 17 % from each other, with a mean deviation of 4.8 %.

### Fatty Alcohols and Acetates

For simplicity, fatty alcohols and their corresponding acetates are described by short names, for instance (*Z*)-11-tetradecen-1-ol is shortened as Z11-14:OH where Z denotes the double bond configuration, 11 the double bond position counting from the carboxylic end, 14 the number of carbons in the chain, and OH denotes the alcohol functional group. The corresponding acetate is denoted as Z11-14:OAc.

The fatty alcohols tested as substrates were obtained from Pherobank or Sigma-Aldrich: 10:OH (Sigma-Aldrich), Z5-10:OH (Pherobank), Z7-10:OH (Pherobank), 12:OH (Sigma-Aldrich), Z7-12:OH (Pherobank), Z9-12:OH (Pherobank), 14:OH (Chemicon), Z9-14:OH (Pherobank), Z11-14:OH (Pherobank), E11-14:OH (Pherobank), Z12-14:OH (Pherobank), E12–14:OH (Pherobank), Z9,E12-14:OH (Pherobank), 16:OH (Sigma-Aldrich), Z9-16:OH (Sigma-Aldrich), Z11-16:OH (Pherobank), E11-16:OH (Pherobank), 18:OH (Sigma-Aldrich), Z9-18:OH (Sigma-Aldrich).

## Results

### Yeast Expressing EaDAcT Produce Acetates When Supplemented with Fatty Alcohols

**Fig. 1:**
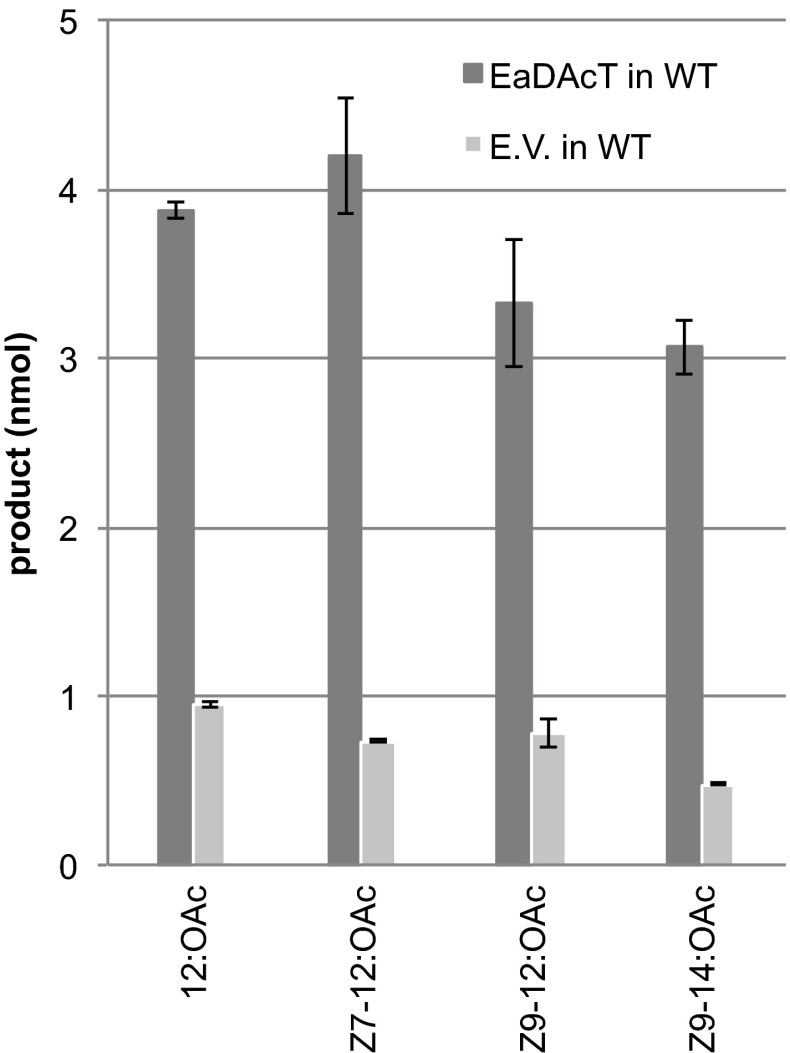
Acetate production in wild-type yeast expressing EaDAcT when supplied with various fatty alcohols. *Bars* show the average amounts of respective acetates recovered from the expression system (yeast cells plus the medium) (*N* = 3). *Error bars* represent SEM

We have previously shown that EaDAcT is capable of acetylating long-chain alcohols in a leaf transient expression system [20]. To further explore the substrate specificity of the enzyme, yeast cells heterologously expressing EaDAcT were exposed to C12 and C14 alcohols in the growth medium. Under these conditions, acetates were synthesized (Fig. 1).

Interestingly, significant quantities of acetates were also formed by yeast containing the empty vector control when the medium was supplemented with the alcohols (Fig. 1), suggesting the existence of endogenous acetyltransferases with specificity towards long-chain alcohols in wild-type yeast.

### Yeast ATF1 is the Only Yeast Enzyme that can Acetylate Long-Chain Fatty Alcohols *In Vivo*

**Fig. 2:**
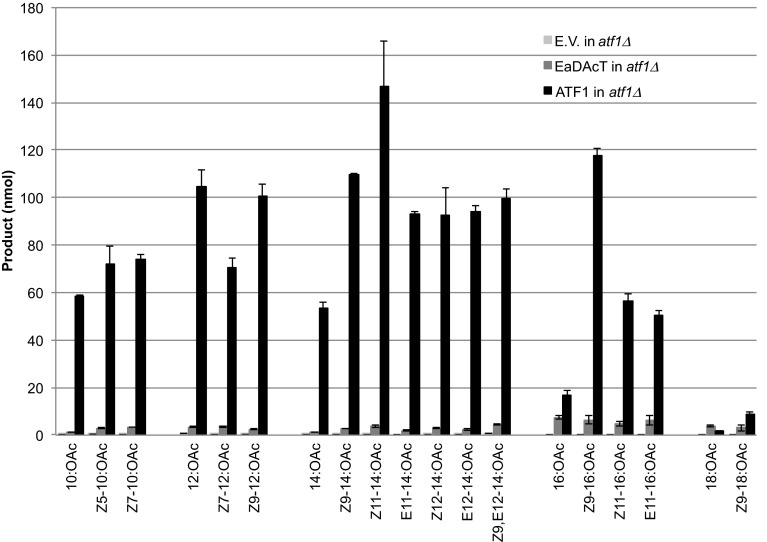
Acetate production in *atf1*Δ yeast with the empty vector (E.V.) or overexpressing ATF1 or EaDAcT, when supplied with various fatty alcohols. *Bars* show average amounts of respective acetates recovered from the yeast cells and the medium (*N* = 3). *Error bars* represent SEM

Given the high long-chain fatty alcohol acetyltransferase background activity, it was of interest to identify the endogenous yeast acetyltransferase(s) responsible for this activity. Previous work has shown that the yeast alcohol acetyltransferase ATF1 can acetylate saturated alcohols up to 16C long when expressed in *E. coli* [24]. We therefore decided to determine whether this enzyme was responsible for the background formation of acetates when supplemented with long-chain fatty alcohols. First, we added 19 different fatty alcohol substrates to the *atf1∆* knockout strain. The *atf1∆* knockout strain produced barely detectable quantities of any long-chain acetates, suggesting that ATF1 is the only yeast enzyme with significant activity towards fatty alcohols of C10 and longer (Fig. 2). To compare the capacity of ATF1 to that of EaDAcT to produce acetates, we expressed either enzyme in an *atf1∆* knockout strain and incubated the cultures with the fatty alcohol substrates. Quantification of the acetates formation provided additional evidence that ATF1 can convert a wide range of fatty alcohols into their corresponding acetates (Fig. 2). Further, the levels of acetates formed in ATF1-expressing yeast were 10–40 times higher than those in yeast expressing EaDAcT, except for 16:OH, 18:OH, and Z9-18:OH which were also poor substrates for ATF1 (Fig. 2).

### Acetates are Secreted into the Growth Medium

**Fig. 3:**
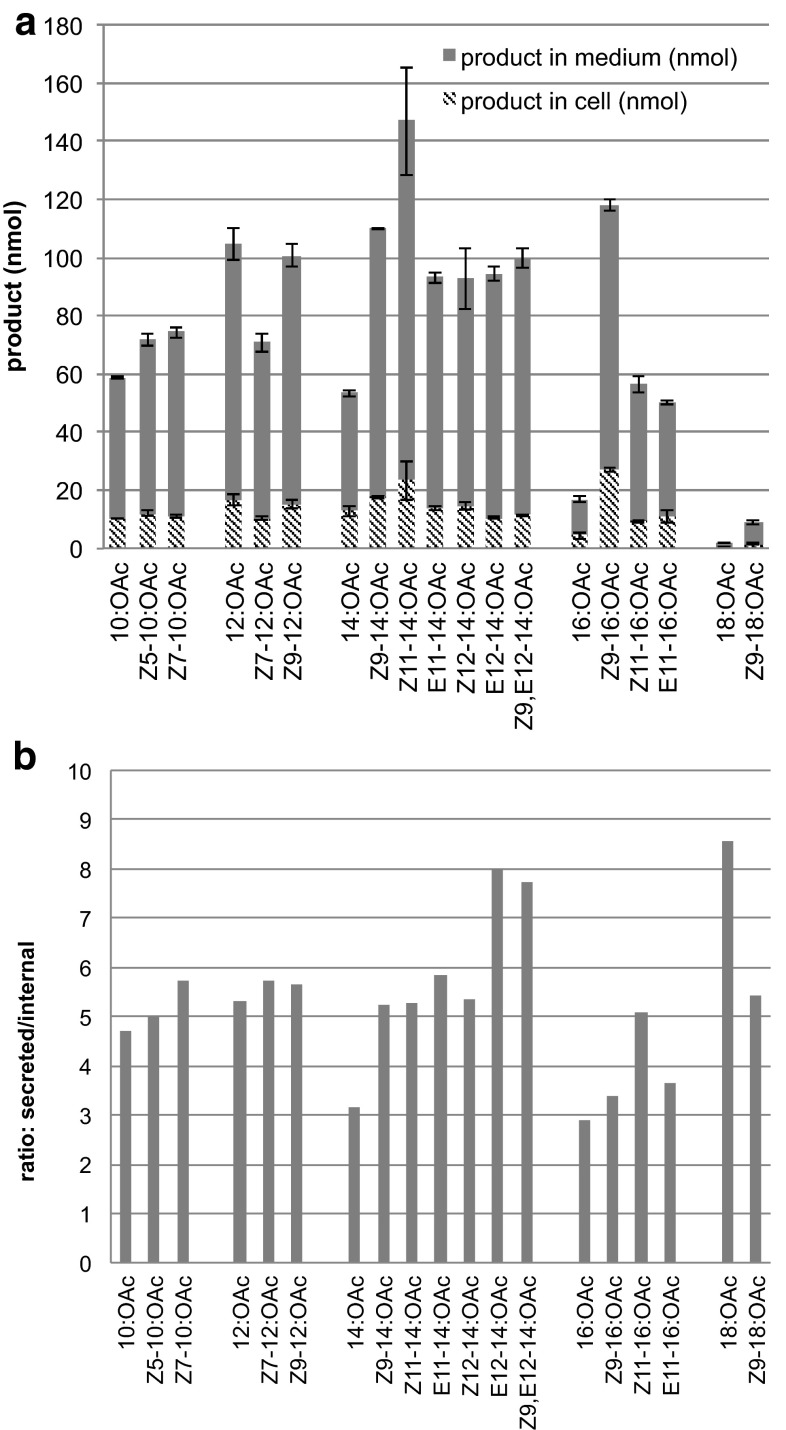
**a** Distribution between medium and yeast cells of acetate produced by yeast overexpressing ATF1 when provided with corresponding fatty alcohol precursors. *Bars* show average amounts of respective acetate (*N* = 3). *Error bars* represent SEM. **b** Relative abundance of secreted versus internal acetates

During the course of the *in vivo* fatty alcohol supplementation experiments, we observed that the acetate products were secreted into the medium (Fig. 3a). Indeed, typically fivefold more acetates were recovered from the media than from the yeast cell pellet. The secretion of the acetates did not appear to be affected by the chain length of the fatty alcohol precursor, as the relative abundance of secreted versus internal product was remarkably constant. However, we observed that the unsaturated acetates were secreted to a larger extent (with the exception of Z9-18:OAc) than their saturated counterpart (Fig. 3b). The export of other acetylated lipids has been previously reported with shorter-chain acetates [22], as well as with acetylated sterols [26].

### *In Vitro* Fatty Alcohol Specificities of EaDAcT and ATF1

**Fig. 4:**
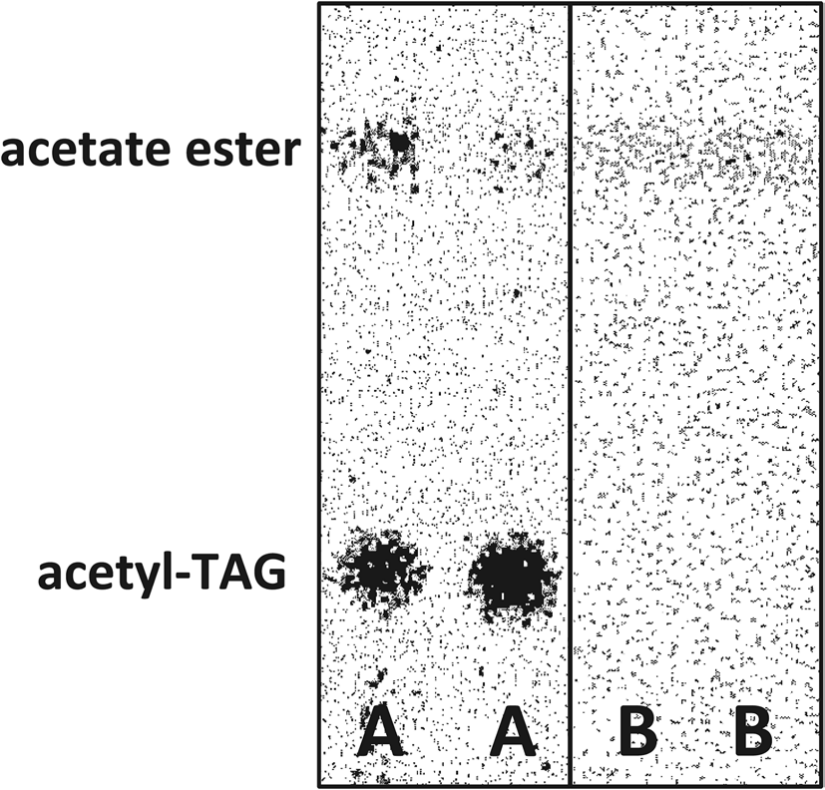
Electronic autoradiogram of TLC separation of lipids from assays of microsomal preparations from yeast expressing *Ea*DAcT incubated with [^14^C]acetyl-CoA and C12 fatty alcohol.* a* Samples from incubations with unwashed microsomes and,* b* samples with acetone-washed microsomes

The production of acetates by feeding various fatty alcohols to yeast expressing acetyltransferases might not reflect true enzyme specificities towards the alcohols since the uptake and thus the *in vivo* concentrations of different alcohols might be very different. Since our ultimate goal is to produce the final pheromone acetates from fed fatty acids by also introducing insect FAD and FAR [21] in addition to the acetylation enzymes, it was important to know how well the amount of acetates formed *in vivo* correlated with the specificities of the acetylating enzymes measured *in vitro.* We therefore isolated microsomes from *atf1Δ* yeast and *atf1Δ* yeast expressing *EaDAcT* or *ATF1*. When microsomes containing EaDAcT were incubated with fatty alcohols and [^14^C]acetyl-CoA, both acetates and acetyl-TAG were formed with over 90 % of the radioactivity residing in the acetyl-TAG (Fig. 4). The latter products result from the acetylation of endogenous diacylglycerides (DAG) contained within the yeast microsomes.

To avoid competition from endogenous DAG and thus better explore the fatty alcohol substrate specificity of EaDAcT, the microsomes were carefully washed with acetone at −80 °C to remove the competing endogenous DAG. Acetone washing of the microsomes totally abolished the formation of radioactive acetyl-TAG but they still retained the capacity to acetylate added long-chain fatty alcohols at about the same extent as the unwashed microsomes (Fig. 4).

**Fig. 5:**
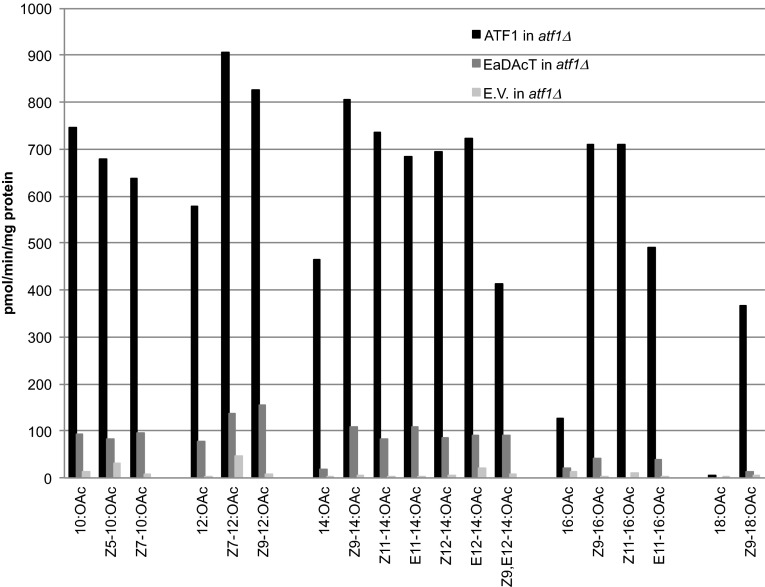
*In vitro* acetyltransferase activity of microsomes prepared from *atf1*Δ yeast, *atf1*Δ yeast overexpressing ATF1 or EaDAcT, and empty vector (E.V.). The *bars* show the average radioactive acetates produced when incubated with the precursor alcohol and [^14^C]acetyl-CoA (*N* = 2)

We then performed *in vitro* assays on acetone-washed microsomal membranes prepared from the *Δatf1* strain and this strain overexpressing ATF1 or EaDAcT. In line with the *in vivo* assay results, the microsomes containing ATF1 possessed much higher activity (up to tenfold) compared to EaDAcT in esterifying the various fatty alcohols (Fig. 5). The activity in microsomes from the *atf1∆* deletion strain was negligible (Fig. 5), suggesting that the other known alcohol acetyltransferases in yeast probably do not acetylate longer alcohols, again consistent with the *in vivo* results obtained (Fig. 2). The specificities measured *in vitro* corroborated well with the amount of acetates formed *in vivo* with various fatty alcohol substrates except for Z9-18:OH. The *in vivo* experiment with this alcohol showed very low activity with this substrate, whereas it was well accepted by ATF1 in the *in vitro* assays. This indicates that Z9-18:OH did not efficiently enter the intact yeast cells. It should be noted that 16:OH and 18:OH were poorly utilized both *in vitro* and *in vivo*.

## Discussion

Fatty alcohol pheromone components can be synthesized by coexpression of two biosynthetic moth-derived genes in yeast cells [21]. Production of acetate requires the action of an acetyltransferase, but until now the acetyltransferase active in moth pheromone biosynthesis has remained elusive. In the endosperm of the burning bush, EaDAcT catalyzes the synthesis of 3-acetyl-1,2-diacyl-*sn*-glycerols (acetyl-TAG) from DAG and acetyl-CoA [27]. We explored another activity of EaDAcT, namely the esterification of long-chain fatty alcohols with acetyl-CoA to produce acetates. This flexibility of EaDAcT substrate specificity is not too surprising given the similarity of the enzyme to the jojoba wax synthase [27]. Interestingly, during the course of this work, we observed significant long-chain fatty alcohol acetyltransferase activity in the yeast empty vector controls (Fig. 1). This background activity was eliminated in an *atf1∆* deletion strain (Figs. 2, 5), suggesting that the alcohol acetyltransferase encoded by *ATF1* is the only endogenous enzyme capable of acetylating longer-chain alcohols to any extent. Previous work has shown that ATF1 is capable of acetylating saturated alcohols up to C14 and C16 [24]. Here, by providing exogenous alcohols not typically produced by yeast, we demonstrate that overexpressed ATF1 can acetylate an even wider range of molecules, including fatty alcohols containing up to 18 carbons, with high efficiency. These results suggest that ATF1 could function as the terminal alcohol acetyltransferase in an engineered pathway for the synthesis of insect pheromones, similar to how EaDAcT has been used previously [20]. Indeed, the much higher activity of ATF1 suggests that it would function as a better fatty alcohol acetyltransferase than EaDAcT for the production of acetate pheromones. Such differences in activity are probably not due to codon bias affecting the expression of a plant gene in a yeast heterologous expression system as EaDAcT shows high DAG acetyltransferase activity under these and similar conditions (Fig. 4) [27].

Under the conditions of both the *in vivo* fatty alcohol feeding studies (Figs. 1, 2) and *in vitro* microsome assays (Fig. 5), ATF1 converted both saturated and monounsaturated fatty alcohols of C10–C14 to their corresponding acetates, whereas saturated C16 and C18 fatty alcohols were poorly acetylated.

This study has identified ATF1 as an acetyltransferase enzyme with high activity toward many fatty alcohol components of insect pheromone. Such an enzyme activity has not previously been characterized. Therefore ATF1 might complete the tool box for the future production of typical moth pheromones from added or endogenous fatty acids in a yeast cell factory without chemical post-processing. The fact that most of the acetate products are secreted into the medium (Fig. 3) may prove convenient for the isolation of moth pheromone components produced in yeast cells.

Multicomponent moth pheromones can be obtained by mixing the products purified from different yeast strains expressing different combinations of genes, similar to previous work using a plant chassis [20]. Although all enzymes for the production of acetate pheromones in heterologous systems might now be available, a future challenge is likely to be to get the activity and the metabolic channeling in the introduced pathway high enough to allow for viable commercial production [21].

## Abbreviations

acetyl-TAG: 3-Acetyl-1,2-diacyl-*sn*-glycerols
ATF1: *Saccharomyces cerevisiae* acetyltransferase I
DAG: Diacylglycerol
EaDAcT: *Euonymus**alatus* diacylglycerol acetyltransferase
FAD: Fatty acyl desaturases
FAR: Fatty acyl reductases
TAG: Triacylglycerol

## References

[1] Ando T (2015) http://www.tuat.ac.jp/~antetsu/List_of_Sex_Pheromones_in_English(2015.12.14).pdf. Accessed 4 Jan 2016

[2] Knipple DC, Rosenfield CL, Miller SJ, Liu W, Tang J, Ma PWK, Roelofs WL (1998). Cloning and functional expression of a cDNA encoding a pheromone gland-specific acyl-CoA ∆11-desaturase of the cabbage looper moth, *Trichoplusia ni*. Proc Natl Acad Sci USA.

[3] Knipple DC, Rosenfield CL, Nielsen R, You KM, Jeong SE (2002). Evolution of the integral membrane desaturase gene family in moths and flies. Genetics.

[4] Liu WT, Jiao HM, O’Connor M, Roelofs WL (2002). Moth desaturase characterized that produces both Z and E isomers of ∆11-tetradecenoic acids. Insect Biochem Mol Biol.

[5] Liu WT, Rooney AP, Xue BY, Roelofs WL (2004). Desaturases from the spotted fireworm moth (*Choristoneura parallela*) shed light on the evolutionary origins of novel moth sex pheromone desaturases. Gene.

[6] Liénard MA, Strandh M, Hedenström E, Johansson T, Löfstedt C (2008). Key biosynthetic gene subfamily recruited for pheromone production prior to the extensive radiation of Lepidoptera. BMC Evol Biol.

[7] Liénard MA, Hagström ÅK, Lassance JM, Löfstedt C (2010). Evolution of multicomponent pheromone signals in small ermine moths involves a single fatty-acyl reductase gene. Proc Natl Acad Sci USA.

[8] Lassance JM, Groot AT, Liénard MA, Antony B, Borgwardt C, Andersson F, Hedenström E, Heckel DG, Löfstedt C (2010). Allelic variation in a fatty-acyl reductase gene causes divergence in moth sex pheromones. Nature.

[9] Hagström ÅK, Liénard MA, Groot A, Hedenström E, Löfstedt C (2012). Semi-selective fatty acyl reductases from four heliothine moths influence the specific pheromone composition. PLoS One.

[10] Morse D, Meighen E (1987). Biosynthesis of the acetate ester precursor of the spruce budworm sex pheromone by an acetyl CoA: fatty alcohol acetyltransferase. Insect Biochem.

[11] Zhao CH, Lu F, Bengtsson M, Löfstedt C (1995). Substrate specificity of acetyltransferase and reductase enzyme systems used in pheromone biosynthesis by Asian corn borer, *Ostrinia furnacalis*. J Chem Ecol.

[12] St-Pierre B, De Luca V (2000) Evolution of acyltransferase genes: origin and diversification of the BAHD superfamily of acyltransferases involved in secondary metabolism. In: Ibrahim R, Varin L, De Luca V, Romeo JT (eds) Recent advances in phytochemistry, vol 34: evolution of metabolic pathways. Elsevier, Oxford, pp 285–315

[13] D’Auria J (2006). Acyltransferases in plants: a good time to be BAHD. Curr Opin Plant Biol.

[14] Itoh N, Slemmon J, Hawke D (1986). Cloning of *Drosophila* choline acetyltransferase cDNA. Proc Natl Acad Sci USA.

[15] Beekwilder J, Alvarez-Huerta M, Neef E, Verstappen FW, Bouwmeester HJ, Aharoni A (2004). Functional characterization of enzymes forming volatile esters from strawberry and banana. Plant Physiol.

[16] Kalscheuer R, Stöveken T, Luftmann H, Malkus U, Reichelt R, Steinbüchel A (2006). Neutral lipid biosynthesis in engineered *Escherichia coli*: jojoba oil-like wax esters and fatty acid butyl esters. Appl Environ Microbiol.

[17] Uthoff S, Stöveken T, Weber N, Vosmann K, Klein E, Kalscheuer R, Steinbüchel A (2005). Thio wax ester biosynthesis utilizing the unspecific bifunctional wax ester synthase/acyl coenzyme A:diacylglycerol acyltransferase of *Acinetobacter* sp. strain ADP1. Appl Environ Microbiol.

[18] Günther CS, Chervin C, Marsh KB, Newcomb RD, Souleyre EJ (2011). Characterisation of two alcohol acyltransferases from kiwifruit (*Actinidia* spp.) reveals distinct substrate preferences. Phytochem.

[19] Fujii T, Ito K, Katsuma S, Nakano R, Shimada T, Ishikawa Y (2009). Molecular and functional characterization of an acetyl-CoA acetyltransferase from the adzuki bean borer moth *Ostrinia scapulalis* (Lepidoptera: Crambidae). Insect Biochem Mol Biol.

[20] Ding BJ, Hofvander P, Wang HL, Durrett TP, Stymne S, Löfstedt C (2014). A plant factory for moth pheromone production. Nat Commun.

[21] Hagström AK, Wang HL, Liénard MA, Lassance JM, Johansson T, Löfstedt C (2013). A moth pheromone brewery: production of (*Z*)-11-hexadecenol by heterologous co-expression of two biosynthetic genes from a noctuid moth in a yeast cell factory. Microb Cell Fact.

[22] Verstrepen KJ, Van Laere SD, Vanderhaegen BM, Derdelinckx G, Dufour JP, Pretorius IS, Winderickx J, Thevelein JM, Delvaux FR (2003). Expression levels of the yeast alcohol acetyltransferase genes ATF1, Lg-ATF1, and ATF2 control the formation of a broad range of volatile esters. Appl Environ Microbiol.

[23] Rodriguez GM, Tashiro Y, Atsumi S (2014). Expanding ester biosynthesis in *Escherichia**coli*. Nat Chem Biol.

[24] Guo D, Pan H, Li X (2015). Metabolic engineering of *Escherichia coli* for production of biodiesel from fatty alcohols and acetyl-CoA. Appl Microbiol Biotechnol.

[25] Banas A, Carlsson AS, Huang B, Lenman M, Banas W, Lee M, Noiriel A, Benveniste P, Schaller H, Bouvier-Navé P, Stymne S (2005). Cellular sterol ester synthesis in plants is performed by an enzyme (phospholipid:sterol acyltransferase) different from the yeast and mammalian acyl-CoA:sterol acyltransferases. J Biol Chem.

[26] Tiwari R, Köffel R, Schneiter R (2007). An acetylation/deacetylation cycle controls the export of sterols and steroids from *S. cerevisiae*. EMBO J.

[27] Durrett TP, McClosky DD, Tumaney WA, Elzinga AD, Ohlrogge J, Pollard M (2010). A distinct DGAT with *sn*-3 acetyltransferase activity that synthesizes unusual, reduced-viscosity oils in *Euonymus* and transgenic seeds. Proc Natl Acad Sci USA.

